# Experimental and theoretically calculated structural data of different iron(II)-terpyridine complexes – validation of theoretical method

**DOI:** 10.1016/j.dib.2024.110423

**Published:** 2024-04-14

**Authors:** Evangelia Athanasopoulos, Marrigje Marianne Conradie, Jeanet Conradie

**Affiliations:** Department of Chemistry, University of the Free State, P.O. Box 339, Bloemfontein 9300, South Africa

**Keywords:** Bis(terpyridine)iron(II), M06/CEP-121G, DFT, Geometry

## Abstract

Experimental structural data for bis(terpyridine)iron(II) and a series of related iron(II) complexes, featuring either substituted terpyridine or tris-azinyl analogues of terpyridine, are presented and analyzed in terms of the Mean Absolute Deviation (MAD) from the average experimental data for each specific complex. The experimental structural data are then juxtaposed with density functional theory (DFT) calculated data obtained using various combinations of DFT functionals and basis sets, with and without the inclusion of Grimme D3 empirical dispersion correction. These diverse computational approaches yield optimized geometries that are subsequently compared against the available experimental structural data to assess their accuracy. The aim is to identify a reliable DFT method for determining the geometries of bis(terpyridine)iron(II) and its related complexes.

Specifications Table

**Table:** 

Subject	Physical and Theoretical Chemistry
Specific subject area	Computational chemistry
Type of data	Table, Image, Analyzed, Filtered, Processed.
Data collection	DFT data were obtained with the Gaussian 16 software programme on the High Performance Computing facility of the University of the Free State
Data source location	Institution: University of the Free State City/Town/Region: Bloemfontein Country: South Africa
Data accessibility	Repository name: Fig share data repository of the University of the Free State (https://ufs.figshare.com/) All data can be accessed at the following link: https://figshare.com/s/16f66aa90a87e650c92a
Related research article	E. Athanasopoulos, J. Conradie, DFT study of the spectroscopic behaviour of different iron(II)-terpyridine derivatives with application in DSSCs, J. Mol. Graph. Model. 129 (2024) 108753. https://doi.org/10.1016/j.jmgm.2024.108753.

## 1 Value of the Data

• This data can be used to visualize the density functional theory calculated optimized structures of different iron(II)-terpyridine complexes
• This data identify the best density functional theory method to reproduce available experimental data of iron(II)-terpyridine complexes
• This data are useful to understand the geometry of structurally unknown iron(II)-terpyridine complexes
• Optimized geometries can be used as starting point to design and optimize new iron(II)-terpyridine complexes

## 2 Background

Accurate and reliable optimized structures obtained through density functional theory (DFT) calculations are essential for further theoretical analysis of various iron(II)-terpyridine complexes, which may include investigations into electronic properties, molecular orbitals, and simulated UV-vis spectra using TDDFT. To achieve this, optimized geometries generated by different DFT approaches and methods must be compared against available experimental structural data. By assessing the Mean Absolute Deviation (MAD) from the average experimental data, DFT methods yielding the smallest deviations can be identified. The provided data aim to facilitate the selection of a dependable DFT methods for determining the geometries of both bis(terpyridine)iron(II) and its related complexes.

## 3 Data Description

**Scheme 1:**
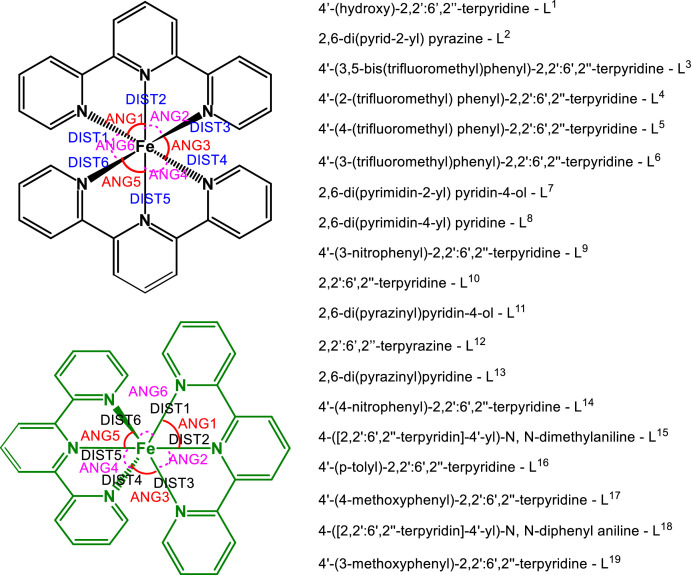
Structure (two different orientations) and complex numbering of [Fe(L^n^)_2_]^2+^ complexes, L = 2,2′:6′,2′'-terpyridine, substituted terpyridine or tris-azinyl analogues of terpyridine, defining bonds and angles around iron(II).

This article describe the dataset of the linked repository https://figshare.com/s/16f66aa90a87e650c92a, where DFT output files using different DFT methods as described in the experimental section, are provided. Scheme 1 shows the structures of the complexes 1 – 19 that were optimized using the different DFT methods. In Table 1, available experimental solid-state X-ray data giving the Fe-N bond lengths (DIST1-DIST6 in Å) and angles (ANG1-ANG6 in °) as defined in Scheme 1, of complexes [Fe(L^n^)_2_]^2+^, n = 1, 6, 9, 10, 16 and 17, are provided. For complexes 10 and 16, where more than one set of structural data are available, the Mean Absolute Deviation (MAD) of the experimental data from the average experimental data, is also provided. In Table 2 the DFT solvent (CH_3_CN) phase calculated Fe-N bond lengths and angles around iron(II), using the different DFT methods, are provided for [Fe(L^10^)_2_]^2+^, complex 10. The MAD of the data obtained by a DFT method, from the average experimental data for 10, is also given, as indicator of the reliability of the calculated data obtained by the specific DFT method. For example, the MAD of the BP86/aug-cc-pVDZ calculated data is 0.34° and 0.004 Å, compared to the average experimental data for 10. In Table 3 the DFT solvent (CH_3_CN) phase calculated Fe-N bond lengths and angles using the M06-D3/CEP-121G method, are given for complexes 1-19. The MAD of the M06-D3/CEP-121 calculated data from the available experimental data is also provided as indicator of the reliability of the calculated data.

**Table 1:** Available experimental solid-state X-ray data for the Fe-N bond lengths (DIST1-DIST6 in Å) and angles (ANG1-ANG6 in °), of complexes [Fe(L^n^)_2_]^2+^, n = 1, 6, 9, 10, 16 and 17. Mean Absolute Deviation (of experimental data from the average experimental data for a specific complex) = MAD. DIST1-DIST6 and ANG1-ANG are defined in Scheme 1.

L^n^	*ANG1*	*ANG2*	*ANG3*	*ANG4*	*ANG5*	*ANG6*	*DIST1*	*DIST2*	*DIST3*	*DIST4*	*DIST5*	*DIST6*	*CSD reference*
L^1^	81.37	80.46	93.70	80.87	80.69	91.39	1.967	1.882	1.978	1.981	1.887	1.973	CAPXUN
L^6^	81.07	81.16	91.78	81.07	81.16	91.78	1.977	1.881	1.984	1.977	1.881	1.984	EWOYAT
L^9^	80.33	80.91	94.24	80.91	80.33	94.17	1.978	1.885	1.971	1.971	1.885	1.978	JEFBUU
L^10^	80.30	80.45	90.92	79.87	82.31	93.00	1.991	1.887	1.954	1.973	1.894	1.967	BETRUO
L^10^	82.02	80.73	92.93	81.21	80.85	90.97	1.970	1.887	1.972	1.978	1.881	1.972	CECCOG
L^10^	80.92	81.00	89.53	81.04	81.35	90.09	1.979	1.889	1.971	1.974	1.868	1.969	CECCOG
L^10^	80.54	80.62	91.92	80.34	80.84	92.23	1.978	1.890	2.001	1.988	1.891	1.984	DANMOU
L^10^	80.91	80.90	93.02	80.76	81.08	89.46	1.974	1.882	1.984	1.983	1.885	1.979	DANMOU01
L^10^	81.17	80.82	92.07	81.08	81.38	92.74	1.977	1.882	1.982	1.971	1.879	1.974	JIPROS
L^10^	81.30	81.10	92.74	81.08	81.12	91.36	1.979	1.885	1.988	1.978	1.890	1.976	JIPROS
L^10^	80.67	81.18	91.22	80.45	81.27	91.50	1.974	1.882	1.967	1.969	1.883	1.972	ULEHUO
L^10^	81.36	81.06	91.74	80.76	81.80	89.43	1.988	1.876	1.977	1.972	1.881	1.970	UYEFIO
L^10^	81.52	80.81	92.51	81.03	81.00	89.74	1.969	1.882	1.973	1.970	1.882	1.965	VIPMUD
L^10^	80.60	80.93	92.33	80.89	80.87	89.24	1.990	1.888	1.990	1.988	1.887	1.981	XENWES
	**81.03**	**80.87**	**91.90**	**80.77**	**81.26**	**90.89**	**1.979**	**1.885**	**1.978**	**1.977**	**1.884**	**1.974**	**average L^10^**
	**0.40**	**0.17**	**0.76**	**0.31**	**0.33**	**1.18**	**0.006**	**0.003**	**0.010**	**0.006**	**0.005**	**0.005**	**MAD L^10^**
L^16^	80.88	80.75	92.30	80.75	80.87	90.67	1.969	1.880	1.981	1.979	1.875	1.982	KOGLAU
L^16^	80.18	80.88	89.52	80.73	80.45	91.31	1.972	1.884	1.975	1.978	1.883	1.986	KOGLAU
L^16^	80.81	81.17	88.73	80.92	81.12	89.67	1.976	1.878	1.981	1.985	1.881	1.985	YEZFAL
L^16^	84.14	79.03	93.49	78.91	84.07	94.12	2.014	1.853	1.998	1.998	1.936	2.012	XOWDES
	**81.50**	**80.46**	**91.01**	**80.33**	**81.63**	**91.44**	**1.983**	**1.874**	**1.984**	**1.985**	**1.894**	**1.991**	**average L^16^**
	**1.32**	**0.71**	**1.88**	**0.71**	**1.22**	**1.34**	**0.016**	**0.010**	**0.007**	**0.007**	**0.021**	**0.010**	**MAD L^16^**
L^17^	80.96	80.63	94.46	80.79	80.76	90.90	1.97	1.88	1.98	1.98	1.88	1.98	JENGES

**Table 2:** Selected DFT solvent (CH_3_CN) phase calculated Fe-N bond lengths (DIST1-DIST6 in Å) and angles (ANG1-ANG6 in °), using the indicated DFT method, of [Fe(L^10^)_2_]^2+^, complex 10. DIST1-DIST6 and ANG1-ANG are defined in Scheme 1. Average = av; Mean Absolute Deviation (of DFT method from the average experimental data for 10) = MAD.

	*ANG1*	*ANG2*	*ANG3*	*ANG4*	*ANG5*	*ANG6*	*DIST1*	*DIST2*	*DIST3*	*DIST4*	*DIST5*	*DIST6*	MAD Angle av	MAD Bond av
Exp. av	81.03	80.87	91.90	80.77	81.26	90.89	1.98	1.88	1.98	1.98	1.88	1.97	0.53	0.006
B3LYP/6-311G(d,p)	80.65	80.65	91.51	80.65	80.65	91.52	2.016	1.911	2.016	2.016	1.911	2.016	0.34	0.006
B3LYP/ aug-cc-pVDZ	80.56	80.56	91.54	80.56	80.56	91.55	2.024	1.917	2.024	2.024	1.917	2.024	0.36	0.006
B3LYP/ cc-pVTZ	80.38	80.38	91.61	80.38	80.38	91.60	2.028	1.918	2.028	2.028	1.918	2.028	0.37	0.006
B3LYP-D3/ CEP-121G	81.39	81.39	91.29	81.39	81.40	91.28	2.001	1.910	2.000	2.001	1.910	2.000	0.34	0.006
B3LYP/ def2tzvpp	80.37	80.37	91.60	80.37	80.37	91.61	2.028	1.918	2.028	2.028	1.918	2.028	0.37	0.006
B3LYP/SDD	81.11	81.11	91.37	81.11	81.11	91.38	2.013	1.910	2.013	2.013	1.910	2.013	0.33	0.006
BP86/ aug-cc-pVDZ	81.18	81.18	91.36	81.18	81.18	91.34	1.976	1.883	1.976	1.976	1.883	1.976	0.34	0.004
BP86/ cc-pVTZ	81.34	81.34	91.31	81.34	81.34	91.30	1.959	1.875	1.959	1.959	1.875	1.959	0.34	0.005
BP86-D3/ CEP-121G	81.97	81.97	91.13	81.97	81.97	91.12	1.957	1.877	1.957	1.957	1.877	1.957	0.37	0.005
BP86/ def2tzvpp	81.00	81.00	91.41	81.00	81.00	91.40	1.979	1.884	1.979	1.979	1.884	1.979	0.32	0.003
BP86/SDD	81.65	81.65	91.21	81.65	81.65	91.22	1.971	1.880	1.971	1.971	1.880	1.971	0.35	0.005
M06/ aug-cc-pVDZ	80.93	80.93	91.42	80.93	80.93	91.43	1.998	1.902	1.998	1.998	1.902	1.998	0.32	0.006
M06/ cc-pVTZ	80.72	80.72	91.49	80.72	80.72	91.50	2.001	1.903	2.000	2.001	1.903	2.001	0.33	0.006
M06/ CEP-121G	81.58	81.57	91.24	81.57	81.57	91.23	1.98	1.89	1.98	1.98	1.89	1.98	0.34	0.005
M06/ def2tzvpp	80.73	80.73	91.48	80.73	80.73	91.49	2.001	1.904	2.001	2.001	1.904	2.001	0.33	0.006
M06/SDD	81.58	81.59	91.22	81.59	81.59	91.23	1.974	1.885	1.974	1.974	1.885	1.974	0.35	0.004
M06-D3/ CEP-121G	81.58	81.57	91.24	81.57	81.57	91.23	1.983	1.895	1.983	1.983	1.895	1.983	0.35	0.004
PBEh1PBE/ aug-cc-pVDZ	80.86	80.86	91.46	80.86	80.86	91.45	1.999	1.899	1.999	1.999	1.899	1.998	0.33	0.006
PBEh1PBE/ cc-pVTZ	80.71	80.71	91.49	80.71	80.71	91.50	2.001	1.899	2.001	2.001	1.899	2.001	0.33	0.006
PBEh1PBE/ CEP-121G	81.35	81.35	91.30	81.35	81.35	91.29	1.994	1.899	1.994	1.994	1.899	1.994	0.34	0.006
PBEh1PBE/ def2tzvpp	80.70	80.70	91.49	80.70	80.70	91.51	2.001	1.899	2.001	2.001	1.899	2.001	0.33	0.006
PBEh1PBE/ SDD	81.37	81.37	91.29	81.37	81.37	91.30	1.987	1.893	1.987	1.987	1.893	1.987	0.35	0.005

**Table 3:** Selected DFT solvent (CH_3_CN) phase calculated Fe-N bond lengths (DIST1-DIST6 in Å) and angles (ANG1-ANG6 in °), using M06-D3/CEP-121G, of complexes 1-19. DIST1-DIST6 and ANG1-ANG are defined in Scheme 1. Average Deviation = AD; Mean Absolute Deviation (of DFT calculated data from the available experimental data) = MAD.

L^n^	*ANG1*	*ANG2*	*ANG3*	*ANG4*	*ANG5*	*ANG6*	*DIST1*	*DIST2*	*DIST3*	*DIST4*	*DIST5*	*DIST6*
L^1^	81.44	81.38	91.30	81.44	81.38	91.26	1.985	1.898	1.984	1.984	1.898	1.984
L^2^	81.39	81.39	91.29	81.39	81.39	91.28	1.988	1.881	1.988	1.988	1.881	1.988
L^3^	81.51	81.52	91.28	81.52	81.51	91.44	1.984	1.890	1.983	1.983	1.890	1.984
L^4^	81.70	81.60	90.91	81.63	81.66	91.07	1.984	1.890	1.981	1.982	1.890	1.983
L^5^	81.50	81.50	91.34	81.50	81.50	91.37	1.984	1.891	1.984	1.984	1.891	1.984
L^6^	81.50	81.55	91.19	81.55	81.50	91.40	1.984	1.890	1.983	1.983	1.890	1.984
L^7^	81.15	81.12	91.38	81.12	81.15	91.34	1.989	1.902	1.989	1.989	1.902	1.989
L^8^	81.33	81.33	91.30	81.33	81.33	91.30	1.983	1.898	1.983	1.983	1.898	1.983
L^9^	81.50	81.54	91.24	81.54	81.50	91.43	1.984	1.891	1.983	1.983	1.891	1.984
L^10^	81.57	81.57	91.24	81.57	81.57	91.23	1.983	1.895	1.983	1.983	1.895	1.983
L^11^	81.44	81.38	91.31	81.38	81.44	91.25	1.976	1.902	1.976	1.976	1.902	1.976
L^12^	81.36	81.36	91.29	81.36	81.36	91.29	1.982	1.889	1.982	1.982	1.889	1.982
L^13^	81.56	81.56	91.24	81.56	81.56	91.23	1.975	1.901	1.975	1.975	1.901	1.975
L^14^	81.52	81.52	91.38	81.52	81.52	91.38	1.984	1.890	1.984	1.984	1.890	1.984
L^15^	81.37	81.37	91.28	81.32	81.32	91.28	1.984	1.892	1.984	1.984	1.891	1.984
L^16^	81.45	81.45	91.34	81.45	81.45	91.33	1.984	1.892	1.984	1.984	1.892	1.984
L^17^	81.44	81.45	91.34	81.45	81.44	91.35	1.984	1.892	1.984	1.984	1.892	1.984
L^18^	81.37	81.37	91.20	81.46	81.46	91.20	1.984	1.890	1.984	1.983	1.891	1.983
L^19^	81.51	81.48	91.42	81.49	81.50	91.25	1.984	1.891	1.983	1.984	1.891	1.984
AD	**0.46**	**0.74**	**1.68**	**0.71**	**0.56**	**0.69**	**0.009**	**0.013**	**0.005**	**0.005**	**0.009**	**0.006**
MAD	**0.27**	**0.17**	**1.16**	**0.17**	**0.28**	**0.68**	**0.005**	**0.004**	**0.003**	**0.003**	**0.003**	**0.003**

## 4 Experimental Design, Materials and Methods

DFT calculations were carried out using the Gaussian 16 software package [1] on molecules treated as singlets (consistent with the reported experimental ground state [2]) and with a charge of 2. Geometry optimizations were performed employing a range of functionals and basis sets as implemented in Gaussian [1], including (i) B3LYP, (ii) M06, (iii) PBEh1PBE, and (iv) BP86, coupled with various basis sets such as CEP-121G, aug-cc-pVDZ, cc-pVTZ, def2tzvpp, and SDD. The Grimme D3 empirical dispersion correction was applied as indicated. These optimizations were conducted in acetonitrile as the solvent, with solvent effects calculated using the implicit solvent Polarizable Continuum Model (PCM) utilizing the integral equation formalism variant (IEFPCM) within Gaussian. The input coordinates for the optimization of the compounds were generated using Chemcraft [3]. Experimental structural crystal data were obtained from the Cambridge Structural Database (CSD) [4], [5], [6].

## Limitations

Not applicable

## Ethics statement

The dataset collected in this study did not involve animals and humans.

## CRediT authorship contribution statement

**Evangelia Athanasopoulos:** Methodology, Visualization, Data curation, Formal analysis, Investigation, Writing – original draft, Writing – review & editing. **Marrigje Marianne Conradie:** Funding acquisition, Writing – review & editing. **Jeanet Conradie:** Supervision, Validation, Methodology, Conceptualization, Funding acquisition, Project administration, Writing – review & editing.

## Data Availability

Iron(II)-terpyridine DFT optimize and TDDFT (Original data) (Fig share data repository of the University of the Free State).
